# A Combinational Strategy upon RNA Sequencing and Peptidomics Unravels a Set of Novel Toxin Peptides in Scorpion *Mesobuthus martensii*

**DOI:** 10.3390/toxins8100286

**Published:** 2016-10-05

**Authors:** Ning Luan, Wang Shen, Jie Liu, Bo Wen, Zhilong Lin, Shilong Yang, Ren Lai, Siqi Liu, Mingqiang Rong

**Affiliations:** 1 Life Sciences College of Nanjing Agricultural University, Nanjing 210095, Jiangsu, China; 15996268076@163.com (N.L.); shenwangzs@163.com (W.S.); 2 BGI-Shenzhen, Shenzhen 518083, China; liujie8@genomics.cn (J.L.); wenbo@genomics.cn (B.W.); linzhilong@genomics.cn (Z.L.); 3 China National GeneBank-Shenzhen, BGI-Shenzhen, Shenzhen, Guangdong 518083, China; 4 Key Laboratory of Animal Models and Human Disease Mechanisms of Chinese Academy of Sciences & Yunnan Province, Kunming Institute of Zoology, Kunming 650223, Yunnan, China; yslzoology@163.com; 5 United Laboratory of Natural Peptide of University of Science and Technology of China & Kunming Institute of Zoology, Chinese Academy of Science, Kunming 650223, Yunnan, China; 6 Sino-African Joint Research Center, Chinese Academy of Science, Wuhan 430074, Hubei, China

**Keywords:** scorpion, peptidomics, toxins, diversity

## Abstract

Scorpion venom is deemed to contain many toxic peptides as an important source of natural compounds. Out of the two hundred proteins identified in *Mesobuthus martensii* (*M. martensii*), only a few peptide toxins have been found so far. Herein, a combinational approach based upon RNA sequencing and Liquid chromatography-mass spectrometry/mass spectrometry (LC MS/MS) was employed to explore the venom peptides in *M. martensii*. A total of 153 proteins were identified from the scorpion venom, 26 previously known and 127 newly identified. Of the novel toxins, 97 proteins exhibited sequence similarities to known toxins, and 30 were never reported. Combining peptidomic and transcriptomic analyses, the peptide sequence of BmKKx1 was reannotated and four disulfide bridges were confirmed within it. In light of the comparison of conservation and variety of toxin amino acid sequences, highly conserved and variable regions were perceived in 24 toxins that were parts of two sodium channel and two potassium channel toxins families. Taking all of this evidences together, the peptidomic analysis on *M. martensii* indeed identified numerous novel scorpion peptides, expanded our knowledge towards the venom diversity, and afforded a set of pharmaceutical candidates.

## 1. Introduction

Venomous animals are the most fascinating species, using poisons or venoms to capture preys or defend themselves. A large number of species belong to the poisonous animal kingdom, such as chordates (reptiles, fishes, amphibians, mammals), echinoderms (starfishes, sea urchins), mollusks (cone snails, octopi), annelids (leeches), nemertines, arthropods (arachnids, insects, myriapods), and cnidarians (sea anemones, jellyfish, corals) [1]. Animal venoms are a rich source of natural compounds that have evolved to have high affinity and selectivity for a diverse range of biological targets. Snake venoms are known to contain 100 unique components while venoms from scorpions, spiders, and cone snails have recently been shown to contain from 300 to over 1000 unique molecular entities [2]. The task to survey the complete diversity of venom is not trivial due mainly to the vast complexity of animal venoms and low resolution of separation techniques. It is this amazing complexity and large number of peptides that pose the greatest challenge to venomics [3]. This task can be deeply approached with two different methods: proteomic/peptidomic and transcriptomic (genomic) approaches [4,5]. The proteomic approach is based on the final product (i.e., the proteins and peptides secreted into the venom), while the transcriptomic approach is based on the genomic recipe book of potential toxins. Combining proteomic and transcriptomic approaches together can be the best way to profile the venom toxin composition.

Approximately 2000 described scorpion species are widely distributed in different ecological niches around the world [6]. The combined methods (proteomic and transcriptomic) have been used to unravel the diversity of scorpion venoms. Complex components of Chinese scorpion venom, such as *Mesobuthus martensii* (also known as *Buthus Martensi Karsch*) [7], *Androctonus bicolor*, and *Heterometrus petersii*, were revealed by chromatography (2-DE, SDS-PAGE, and RP-HPLC) with mass spectrometry (ESI-Q-TOF MS and MALDI-TOF MS), and transcriptomics [8,9,10,11]. Two-hundred twenty-seven proteins unambiguously have been identified from *M. martensii*. Among those proteins, only 50 peptides are toxins and the others are enzymes or cell-associated proteins. In *Androctonus bicolor*, there are 103 novel putative peptides and only 16 were identified at the protein level.

*M. martensii* is one of traditional medicine in China. Fifty toxins have been identified in the venom of this species. The amount of known/identified peptides is far lower than the estimated amount, implying a need for deep analyses in venomics [12]. Here, peptidomic and transcriptomic approaches were employed to fully explore the deep diversity of the venom peptides from this scorpion.

## 2. Results

### 2.1. Venomics of the Scorpion Gland

**Figure 1:**
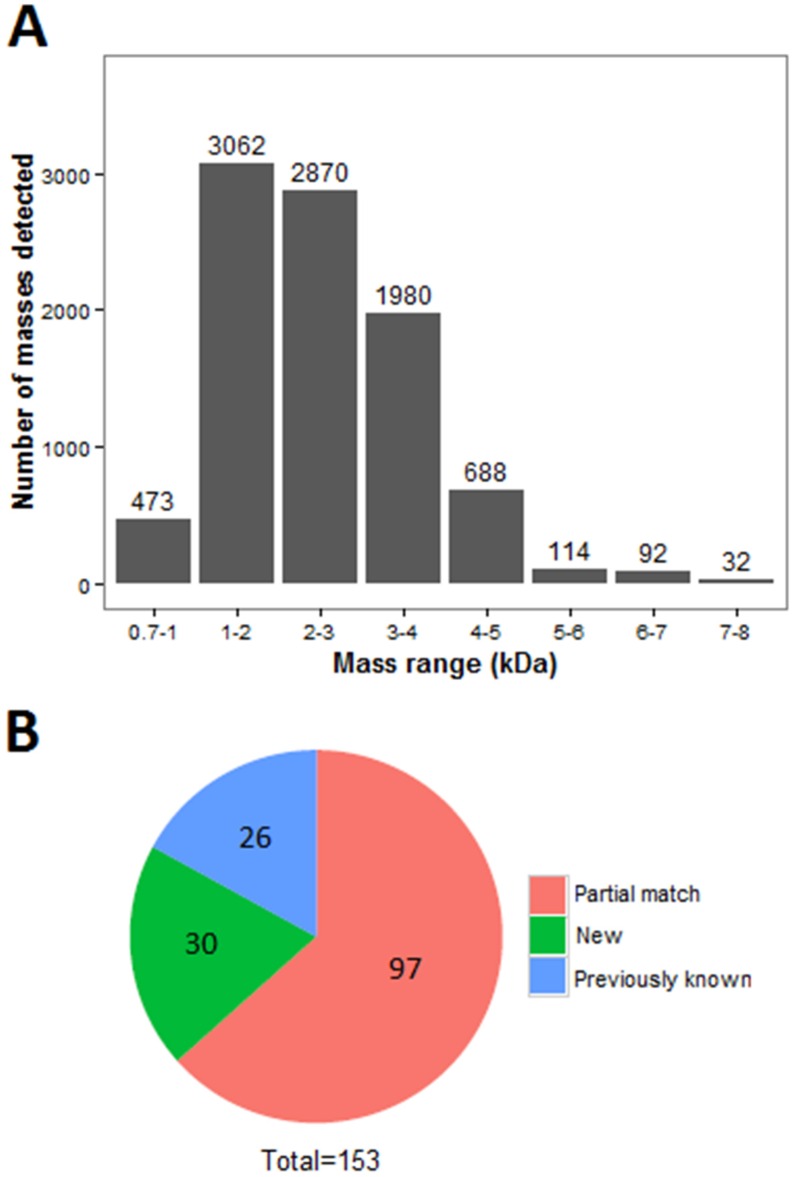
Molecular weights and number of peptides (peptide precursor ions detected by LS/MS). (**A**) The molecular weights of fragments from scorpion venom. The *x*-axis represents the molecular weight (10^3^) and the *y*-axis are the amounts of fragments; (**B**) The classification of identified proteins by BLAST results. The known proteins are in blue, partially-matched proteins are red, and novel proteins are green.

De novo assembly of RNA-Seq data using Trinity software generated 79,804 transcripts. Proteomic analysis of the scorpion *Mesobuthus martensii* has been reported before and proteins from this species were isolated by 2-DE, SDS-PAGE, and RP-HPLC [8]. In order to focus on peptides, fractions with mass weight less than 10 KD (identified by MALDI-TOF MS) were analyzed to uncover the complexity of the venom. The small peptides were detected on a Prominence Nano-HPLC system coupled with Q-Exactive. As shown in Figure 1, most of the fragments (peptide precursor ions detected by LS/MS) have molecular weights from 1000 to 5000 Da, and only a few fragments are larger than 5000 Da. This study focused mainly on peptide toxins and larger proteins (> 10 KD) were filtered manually. The molecular weight of peptides ranges from 0.7 to 8 KD (Figure 1A). The number of peptides with molecular weight from 0.7 to 1, 1 to 2, 2 to 3, 3 to 4, 4 to 5, 5 to 6, 6 to 7, and 7 to 8 KD were 473, 3062, 2870, 1980, 688, 144, 92, and 32, respectively. This indicates that most of peptides are small fragments. Finally, 708 unique peptides were identified and 153 proteins were identified by the two analysis methods (Tables S1 and S2). Among the identified proteins, 26 toxins showed the same sequence to BLAST results, 97 proteins were partially matched, and 30 were novel venom proteins unknown before (Figure 1B).

**Figure 2:**
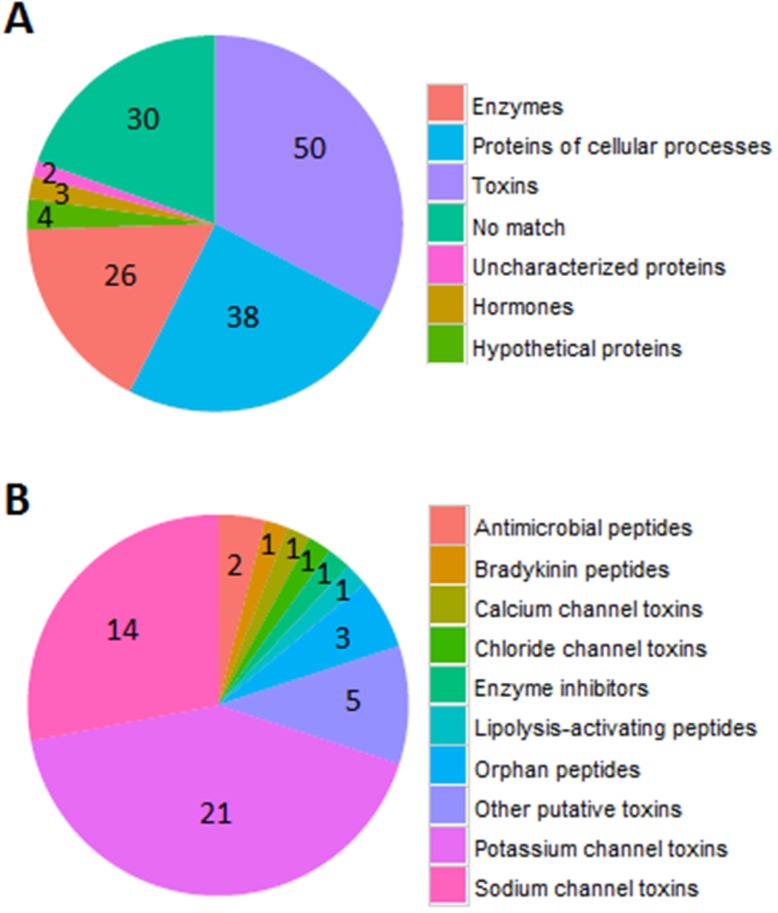
The function of identified toxin proteins. (**A**) The different functions of proteins, including toxins, cellular process proteins, enzyme, hypothetical proteins, hormones, uncharacterized and unknown proteins, are in different colors; (**B**) The number of different functional proteins from 50 toxins. 21 proteins target potassium channels, 14 proteins have activity on sodium channels, 5 proteins are putative toxins, three are orphan peptides, 2 are antimicrobial peptides, 1 is a calcium channel toxin, 1 is a lipolysis-activating peptide, 1 is bradykinin, 1 is a chloride channel toxin, and 1 is an enzyme inhibitor.

The 26 known and 97 partially-known proteins were categorized to different function groups. Among these proteins, 50 belong to toxins, 38 to cellular process proteins, 26 to peptide fragments of enzymes, three to hormones, four to hypothetic proteins, and two to previously uncharacterized proteins (Figure 2A). Toxins were the major components in venom, which were further divided into 11 function groups, including 21 potassium channel inhibitors, 14 sodium channel toxins, 2 antimicrobial peptides, 1 chloride channel toxin, 1 lipolysis-activating peptide, 1 bradykinin, three orphan peptides, and 5 putative toxins (Figure 2B).

### 2.2. Identification of Peptides by LC-MS/MS Combined with RNA-Seq Data

**Figure 3:**
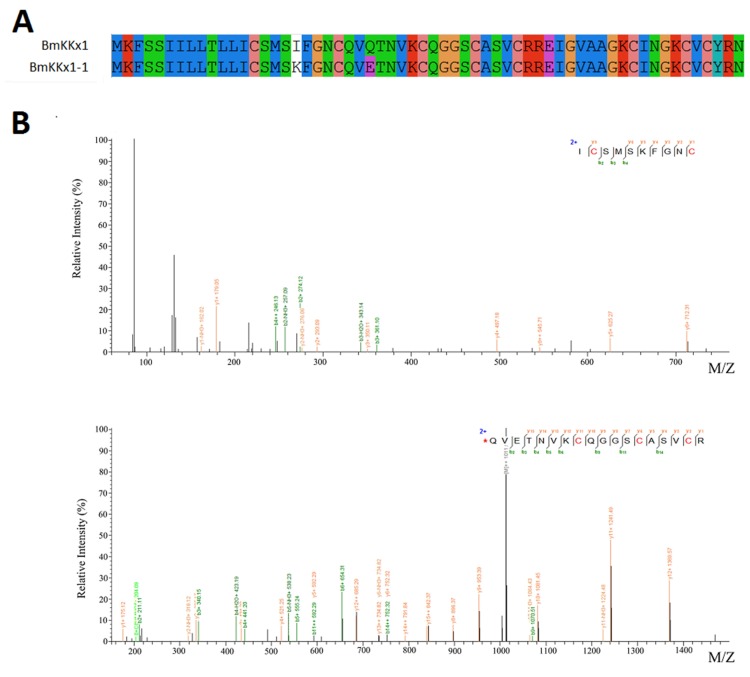
Identification of a novel toxin BmKKx1-1. (**A**) Sequence alignment of two homologs. The alignment of sequences was based on the results of MEGA; (**B**) Seven y-ions and three b-ions were identified from the upper MS/MS spectra, and 15 y-ions and eight b-ions were identified from the lower MS/MS spectra. The spectral images were generated using pLabel (http://pfind.ict.ac.cn/software/pLabel/index.html).

Some scorpion toxins have been identified by LC/MSMS combined with RNA-Seq data. BmKKx1-1 showed only two different residues (the 18th (I to K) and 25th (Q to E) residues) in the mature fragments compared to BmKKx1. It was very interesting to find that the mature peptide of BmKKx1-1 begin from the 13th residue and formed four disulfide bridges (Figure 3). These results were different from the previous results in that BmKKx1 contained a 22-residue signal peptide and a 38-residue mature peptide with three disulfide bridges [13]. Combining peptidomic and transcriptomic methods was a good way to identify the initiation site of mature peptides.

### 2.3. Sodium Channel Toxins

**Figure 4:**
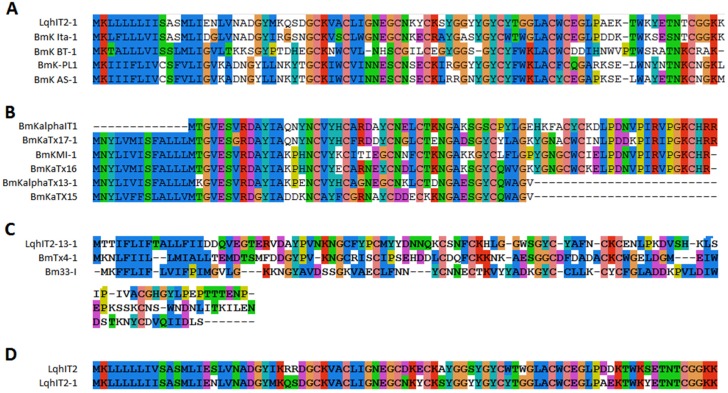
Alignment of sodium channel toxins from *M. martensii*. All sodium channel toxins were aligned by similarity. Sequences of sodium channel toxins belong to family 1 (**A**) and family 2 (**B**); (**C**) Three other sodium channel toxins identified from scorpion venom with low similarity were found; (**D**) Sequence alignment of LqhIT2-1 and LqhIT2.

Fourteen sodium channel toxins were found in this study. Most of them were long chain toxins and contained four disulfide bridges. Eleven toxins obviously belonged to two different families by sequence similarity. Six peptides were α-toxins (sodium channel toxins alpha-toxins (α-toxins)) and eight peptides were beta-toxins (β-toxins) by function effect [14]. One toxin (BmKAs1) has been identified before and four (BmK ITα-1, BmK BT-1, BmK-PL1, and Lqh IT2-1) toxins showed amino acid sequence similarities to BmKITa, BmK BT BmK AS, and Lqh IT2 (Figure 4A) [15,16,17]. Although Lqh IT2 and Lqh IT2-1 were isolated from scorpion in the Middle East and in China, a high conservation (84% identity) was found between the two toxins (Figure 4D). The second family included six toxins. BmKaIT1, BmKaTx16, and BmKaTX15 were alpha-insect toxins reported in previous works [18]. The sequence of other three (BmKaTx17-1, BmKaTx13-1, and BmKI-1) were similar to BmKaTx17, BmKaTx13, and BmKI (Figure 4B) [18,19]. Although the two toxin families showed low sequence similarity to each other, the cysteine framework of the first family (C_3_-C_6_-C_3_-C_9_-C_6_-C_1_-C_16_-C) and second family (C_3_-C_5_-C_3_-C_9_-C_9_-C_1_-C_14_-C) exhibited high similarity, implying the high conservation of this cysteine framework in scorpion toxins. Three other sodium channel toxins had no similarity to each other. Bm33-I was an insect sodium channel toxin known before [20]. BmTX4-1 showed 91% similarity to BmTX4 from *Mesobuthus eupeus*.

### 2.4. Potassium Channel Toxins

**Figure 5:**
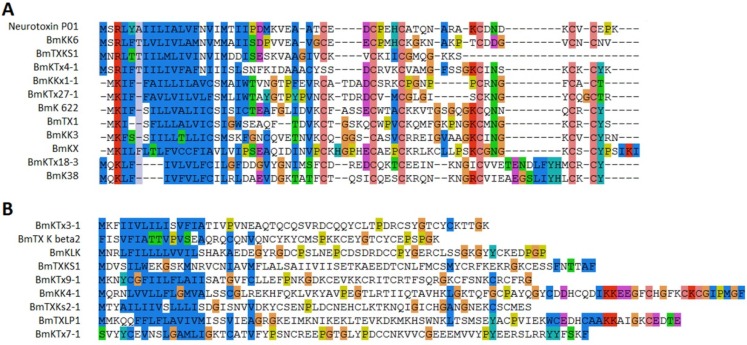
Alignment of potassium channel toxins from *M. martensii*. (**A**) Twelve potassium channel toxins aligned based on sequence similarity and all toxins had six cysteines; (**B**) Other identified potassium channel toxins.

Scorpion venom contained many toxins targeting potassium channels. Twenty-one inhibitors were detected in our study, including 11 known and 10 novel peptides. Most of them were medium-long chain peptides with six cysteines forming three disulfide bridges, while two toxins (BmTXKbeta2, BmKLK) were previously-known long chain potassium channel toxins. Four known (BmTx3B, BmTX1, BmKK3 and BmKX) and two novel (BmKKx1-1 and BmKTx27-1) potassium channel toxins exhibited high similarity to each other and were considered as α-KTxs (Figure 5A). The novel potassium toxin BmKKx1-1 was a mutant to BmKKx1 from the same species, but BmKTx27-1 showed 86.36% similarity to toxin Tx771 from another scorpion species *Buthus occitanus Israelis* [21]. Three toxins (BmKK6, neurotoxin P01 and BmKTx4-1) showed high similarity in the signal peptide and was classified to the same family. Toxin BmKTx4-1 was a novel toxin and exhibited 96.55% similarity to pMeKTx4-1 from *M. eupeus*. It is very interesting that the other five novel toxins (BmKTx7-1, BmKTx30-1, BmKTx18-3, BmKTx9-1 and BmKTx3-1) also showed similarity to the toxins from *M. eupeus* (Figure 5).

### 2.5. Other Functions

Scorpion venom also contained many other functional toxins. One calcium channel toxin (BmCa1), one chloride channel toxins (Bm12-b), two antimicrobial peptides (marcin-18, BmKn1), and one bradykinin-potentiating peptide (BmKbpp) were detected from *M. martensii* (Table 1) [10,22]. A novel lipolysis-activating peptide (BmLVP2-alpha) has large variation in the signal peptide compared to BmKBT, but only four residues differed in the mature peptide between two peptides [23]. A novel inhibitor of nitric oxide (NO) synthase Makatoxin-2-1 showed amino acid sequence similarities to MKTXII. Three orphan peptides (BmOp-1, BmOp-2 and BmOp-3) exhibited high similarity to AbOp-12 (95%), AbOp-25 (87%), and AbOp-3 (84%) from *Androctonus bicolor* (Table 1). Five peptides (BmPt1- BmPt5) were considered as potential toxins. Those toxins showed similarity to other scorpion species, except BmPt3 and BmPt4. BmPt1 showed 99% identity to venom toxin meu VNP2 from *M. eupeus*. A high identity (91%) was found between BmPt2 and venom protein VP5 from *Odontobuthus doriae*. BmPt5 showed 67% identity to U1-buthitoxin-Hj1b from *Hottentotta judaicus* (Table 1).

### 2.6. Novel Venom Peptide

Peptide/protein sequences were searched on the NCBI nr database using the online BLAST with an *e*-value cutoff set to <10^−5^. Thirtypeptides showed no similarity to any known proteins, implying those peptides were novel. Both long- and short-chain peptides were found in this new group and may represent the sequence diversity of unknown peptides of scorpions. BmVt18 contained 60 residues and the mature peptide sequence was PSHRILYNIKIGLYCFKFCLYRRKTAYSIICNCYLNTLRSLR. Four cysteines were found in the mature sequence, indicating this peptide may be a potential toxin. Most of the novel venom peptides did not resolve in full sequences, which may be due to the low abundance in venom (Table 2).

### 2.7. Amino Acid Variability in Peptide Toxins

**Figure 6:**
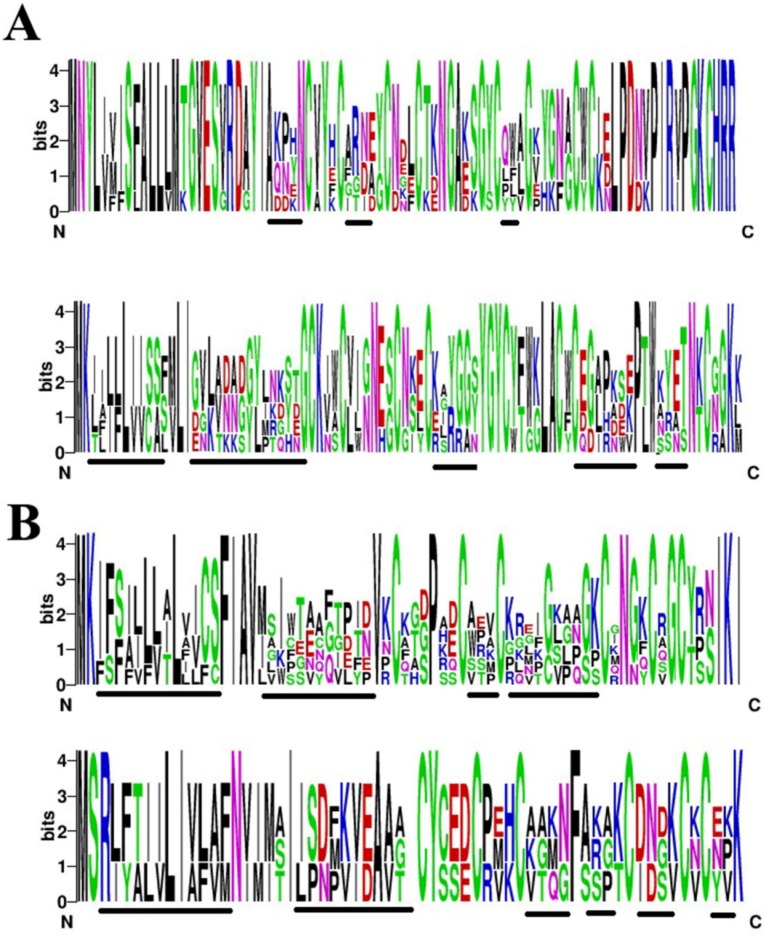
Sequence logos of toxins from scorpion. (**A**) Sodium channel toxins from family 1 (upper) and family 2 (lower); (**B**) Potassium channel toxins from Figure 5A. Each logo consists of stacks of letters and the overall height of each stack indicates the sequence conservation at that position (measured in bits).

Peptide toxins from scorpion were rich in sodium and potassium channel toxins. Two sodium channel toxin families and two potassium channel toxin families were found in this work. Although the toxins belong to the same families, the sequences seemed variable in many regions. In order to analyze the diversity of toxins, we used WebLogo, a web-based application designed to generate sequence logos to compare conservation and variety of toxin families. As showed in the Figure 6A, the first sodium channel toxin family had high conservation except for charged residues in the middle of their peptide sequences. Another sodium channel toxin family exhibited a diversity of signals and propeptides. Three highly variable positions (marked with underline) containing hydrophobic and charged amino acid (Figure 6A). Scorpion venom was very rich in potassium channel toxins. As shown in Figure 6B, two families of potassium channel toxins exhibited a high variety in signal peptides and mature peptides, except six cysteine residues (Figure 6B).

## 3. Discussion

Scorpion venom contains a larger number of peptide toxins, which show diversity in structure and biological activity. Hundreds of proteins have been reported, including the inhibitors of the potassium channel, sodium channel, and chloride channel, antimicrobial peptides, lipolysis-activating peptide, and bradykinin [8]. Such a wide range of peptides and proteins with different biological functions make scorpion venoms a valuable source of new compounds both for use in basic research and for the development of new medicines [24]. It also stimulates us to thoroughly investigate the diversity of the scorpion toxins of *M. martensii*.

In the past years, transcriptomic analysis was the most useful method to identify the sequences of peptide toxins from venom glands. The signal peptides, propeptides, and mature peptides of those toxins were determined by bioinformatics. BmKKx1 was first found from the cDNA library and showed 84% identity to BmTx3 [25]. It was thought that the mature sequence of BmKKx1 was QVQTNVKCQGGSCASVCRREIGVAAGKCINGKCVCYRN. High accuracy and throughput mass spectrometry provides a powerful platform to investigate the trace peptide. In this study, a fragment (ICSMSKFGNC) of a novel peptide BmKKx1-1 was determined at the protein level by mass spectrometry. This result indicates that BmKKx1-1 should start at the 13th residue and contain eight cysteines. Thus, peptidomics combined with transcriptomics, may be an accurate method to obtain the correct mature sequence of undetermined toxins.

Thousands of peptides fragments were found by mass spectrometry, and the molecular weights of most fragments ranged from 1000 to 5000 Da. The total number of peptides/proteins identified in this work was 153, indicating that some of the toxins may contain more than one fragment, such as BmKKx1-1 (Figure 2). Peptidomic methods could unsuspectedly determine partial sequences of peptides, but it seems difficult to get the full sequence of long toxins. Peptidomics combined with transcriptomics may be a good way to obtain the correct and full sequence.

Although larger proteins were eliminated in the process, some proteins, such as those involved in cellular processes, enzymes, and hormones, were still identified in the scorpion venom. This may be caused by alternative cleavage of the larger proteins and the fragments existed in the venom. Among the identified toxins, 26 toxins, including sodium channel, potassium channel, and calcium channel toxins, have been detected before. The other 127 proteins were identified for the first time. The novel toxin BmKK4-1 showed 98% similarity to the known scorpion toxins BmKK4, and only one residue is different between the two toxins [26]. The similarity of other novel 96 toxins to known toxins ranged from 42% to 98%. Most of the similar toxins were identified from the same scorpion speciies, *M. martensii*. However, BmTX4-1 exhibits 92% similarity to BmTX4 from *M. eupeus*, and four other potassium channel toxins (BmKTx30-1, BmKTx18-3, BmKTx9-1 and BmKTx3-1) are also proved to show amino acid sequence similarities to toxins from *M. eupeus* [27], suggesting that the two scorpions had a closer relative relationship. The novel toxins found increase the diversity of the scorpion toxins from *M. martensii*. The highly-conserved toxins (LqhIT2-1 and LqhIT2, BmTX4-1 and BmTX4, BmKTx30-1 and BmKTx30, BmKTx18-3 and BmKTx18, BmKTx9-1 and BmKTx9, and BmKTx3-1 and BmKTx3) from *M. martensii*, *M. eupeus*, and *Leiurus quinquestriatus hebraeus* may provide an example that those toxins under the heavy constraints of negative selection [28].

Highly-conserved and variable regions were obviously found among 24 toxins, which belong to two sodium channel and two potassium channel toxin families. Cysteines are highly-conserved in all toxin families, and other residues are varied in mature peptides. Sodium channel toxins are more conserved than potassium channel toxins in mature regions. The first sodium channel toxin family only shows small changes in three short regions (Figure 6A), while most of the residues in the mature region of the potassium channel toxin family are variable. Those results imply high evolutionary changes and diversity in scorpion venom.

## 4. Conclusions

In summary, we used peptidomics combined with transcriptomics to uncover the diversity of toxins from scorpion *M. martensii*. Our data indicated that at least 127 novel proteins were found in this scorpion and broadened our knowledge about the diversity of venom. The greatly diverse toxins from *M. martensii* will provide candidates for medical pharmaceuticals.

## 5. Materials and Methods

### 5.1. Venom Collection

Adult *M. martensii* scorpions (both sexs, *n* = 100) were purchased from Shandong Province, China. Crude venom of the scorpion was collected manually by electrical stimulation of the telson using a 25–50 V and 15–40 Hz pulse current. Protease inhibitor cocktail (Roche, Basel, Switzerland) was added to crude venom according the manufacturer’s instructions [29]. Crude venoms were stored at −80 °C until further use.

### 5.2. Transcriptome Sequencing

Total RNA was extracted from the venom glands of 10 scorpions using Trizol reagent (Invitrogen, Carlsbad, CA, USA) and purified using RNeasy Animal Mini Kit (Qiagen, Valencia, CA, USA) according to the manufacturer’s instructions. RNA integrity number (RIN) values were measured for all samples. The RIN of *M. martensii* was 7.7.

mRNA was isolated from the total RNA using poly-T oligo-attached magnetic beads (Invitrogen, Carlsbad, CA, USA)and was fragmented into short fragments (200–700 bp) using divalent cations under elevated temperature. Short fragments were purified and resolved with EB buffer (Qiagen, Valencia, CA, USA) for end reparation and single nucleotide A (adenine) addition. After that, the short fragments were connected with adapters. Suitable fragments were selected as templates for PCR amplification and abundance normalization. During the QC steps, an Agilent 2100 Bioanaylzer (Agilent, Santa Clara, CA, USA) and an ABI StepOnePlus Real-Time PCR System (ABI, Waltham, MA, USA) were used in quantification and qualification of the sample library. At last, the library was sequenced using Illumina HiSeq™ 2000 (Illumina, San Diego, CA, USA). After sorting, cleaning and trimming of the reads, sequence assembly was carried out using Trinity (trinityrnaseq_r2012-06-08) [30]. The FASTAQ data has been deposited to NCBI (accession: PRJNA341817).

### 5.3. Venom Sample Preparation

Crude venom was suspended in 8 M urea in 0.1 M Tris-HCl (Sigma, St. Louis, MO, USA), pH 8.5, and centrifuged at 16,000 g for 10 min at 4 °C. Protein concentrations were detected using Bradford reagent (Sigma, St. Louis, MO, USA). Denatured proteins and peptides were reduced with 10 mM DTT (Amresco, Solon, OH, USA) at 56 °C for 1 h. After cooling to room temperature, the proteins were alkylated with 55 mM IAA (Sigma, St. Louis, MO, USA) in the dark at room temperature for 45 min [31].

### 5.4. Solid-Phase Extraction of Venom Peptides

The alkylated venom solution was diluted with 8 M urea to 1 mL, and then fractionated in a Strata-X C18 (Phenomenex, Torrance, CA, USA) column previously conditioned with methanol. After loading the venom solution the column was washed with 0.1% FA (J&K, Beijing, China) in 5% ACN (Thermo Fisher scientific, Waltham, MA, USA) and eluted with 80% ACN. The eluates were dried in a SCANVAC (LaboGene, Lynge, Denmark) concentrator. The dried venom eluates were stored at −20 °C for further analysis.

Venom peptide concentrations were determined using a NanoDrop A280 system (Thermo Fisher Scientific, Waltham, MA, USA), and peptide amounts in the eluates were estimated based on absorbance at 280 nm using the nanodrop system (Thermo Scientific) [32]. HILIC separation was performed on a 20 AD HPLC system (Shimadzu, Tokyo, Japan) using ZIC®-HILIC 250 × 4.6 mm, 5 μm, 200® PEEK HPLC Column SeQuant(TM). Briefly, 200 μg mixed peptides were diluted with buffer A and loaded onto the HILIC column at 0.5 mL/min. Buffer A was composed of 80% can and 0.1% TFA (J&K, Beijing, China), and buffer B was composed of 0.1% TFA. After a 10 minute post injection hold at 100% A, peptides were eluted with a gradient from 0 to 40% B in 10 min followed by 40% to 90% B in 2 min and a 10 min hold to end. The absorbance at 214 nm was monitored, and a total of 10 fractions were collected along the gradient. Collected fractions were dried in a SCANVAC concentrator and stored at −20°C until use for nano LC-MS/MS analysis.

### 5.5. Nano LC-MS/MS Analysis

Liquid chromatography and mass spectrometry were performed on a prominence nano-HPLC system (Shimadzu, Tokyo, Japan) coupled with Q-Exactive (Thermo Fisher Scientific, Waltham, MA, USA). The peptides were separated by nano-LC on an in-house packed Ultimate XB-C18 column (3 μm, 12 cm × 75 μm, Welch Materials) at a flow rate of 300 nl/min. Each fraction was dissolved in 0.1% FA, then injected and eluted using a gradient of 5%–30% solvent B (95% acetonitrile, 0.1% FA) over 40 min. The mass spectrometry was operated in data-dependent mode, automatically switching between MS and MS2 acquisition. Survey full-scan MS spectra (*m*/*z* 350–1800) were acquired in the Orbitrap with a resolution of 70,000. The 20 most intense ions were sequentially isolated and fragmented by high energy dissociation (HCD). Peptides with unassigned charge states, as well as less than +2, or more than +6, were excluded from fragmentation. Fragment spectra were recorded in the Orbitrap mass analyzer with resolution of 17,500. The dynamic exclusion was enabled with repeat count two, and an exclusion duration 8 s.

### 5.6. Peptide Identification and Bioinformatic Analysis

The raw MS/MS data were converted into MGF format by Proteome Discoverer 1.2 (Thermo Fisher Scientific, Waltham, MA, USA). The assembled transcript sequences were translated to protein sequences using PGA with the six frame method as the searching database [33]. The MS/MS spectra were searched by Mascot (v2.3.02, MatrixScience; London, UK) against the database. Mascot parameters were set as follows: no enzyme selected; fixed modifications of Carbamidomethyl (C); variable modifications consisting of Gln->pyro-Glu (N-term Q) and Oxidation (M); peptide charge, 2+, 3+, and 4+; 10 ppm of peptide mass tolerance; 0.05 Da of fragment mass tolerance. The automatic Mascot decoy database search was performed. The Mascot results were processed by IQuant [34] utilizing MascorPercolator to re-score the peptide spectrum matches (PSMs) [35], and false discovery rates (FDR) were obtained using the target-decoy strategy with a peptide-level FDR equal to or less than 0.01. Finally, the identified peptide sequences were assembled into a set of confident proteins using the Occam’s razor approach implemented in IQuant.

Annotation of identified protein sequences was searched against the NCBI NR database with an *e*-value cutoff set to <10^−5^ for comparison using the online Blast (http://blast.ncbi.nlm.nih.gov/Blast.cgi), and optimized manually. The resulting gene superfamily sequences were further aligned using the ClustalW alignment tool in the MEGA package (version 6.06) [36]. Aligned toxin sequences were used to create sequence logos by WebLogo (version 2.8.2, University of California, Berkeley, CA, USA, 2016) [29,30,37,38].

### 5.7. Ethics Statement

The Animal Care and Use Committee of The Kunming Institute of Zoology, Chinese Academy of Sciences, reviewed and approved all of the procedures used in this study (2015-249). All experiments were performed according to good practices of laboratory animal management.

## Figures and Tables

**Table 1:** Scorpion toxins of other function.

Name of Toxin	Identity	Evalue	Function	Sequnce
BmCa-1	100	2.76E-41	Calcium channel toxin	MNTFVVVFLLLTAILCHAEHALDETARGCNRL NKKCNSDGDCCRYGERCISTGVNYYCRPDFGP
Bm12-b	100	3.14E-36	Chloride channel toxin	MKFLYGIVFIALFLTVMFATQTDGCGPCFTTD ANMARKCRECCGGNGKCFGPQCLCNRE
Marcin-18	100	9.09E-48	Antimicrobial peptide	MQFKKQLMVIFLAYFLVVNESEAFFGH LFKLATKIIPSLFRRKNQRSRSIMKRDLE NLFDPYQRNLELDRLLKQLP
BmKn1	100	8.51E-43	Antimicrobial peptide	MKSQTFFLLFLVVLLLAISQSEAFIGAVAGLLSKIFGK RSMRDMDTMKYLYDPSLSAADLKTLQKLMENY
BmOp-1	95.38	3.48E-34	Orphan peptide	MKPRTFLVLFLVCILVMDAVVARRCGGNRRRKVI KMVRKLRPLVKVIRIIKKIRGRAPCGGSRLT
BmOp-2	86.59	9.63E-46	Orphan peptide	MNKSLIILIVAIVVLSLWSAAEARQRRCQVYC SNSPKGCQVRCSWSFGKREIADTVEDFPGKL DEDDEAILKKLAIEELKNE
BmOp-3	83.76	5.15E-59	Orphan peptide	MKTTGIILFVCAIIYSLYLEAESASIHSSEL QSNFHKLRKRQIHGRFGDYIPRGDLAQG KVSTGVEMFFPVDDDGEETRDTDDDGEQ TSRSEDDDDGLTSRRAAPRERHVNCNKRHY
BmLVP2-alpha	85.71	8.46E-59	Lipolysis-activating peptide	MMTLALFGIIFTLFSLIGSIHGDDEPGDYPTNVYGNK YYCTILGENEYCRKICKLHGVSYGYCYNSRCWCEY LEDKDVTIWNAVKNHCTNTLLYPNGK
Bpp BmK3	100	1.06E-44	Bradykinin-potentiating peptide	MNKKTLLVIFFVTMLIVDEVNSFRFGSFLKKVWKSKL AKKLRSKGKQLLKDYANKVLNGPEEEAAAPAERRR
Makatoxin-2-1	90.91	6.3329E-39	Inhibitor of nitric oxide (NO) synthase	MNYLIVISFALLLMTGVESGRDAYIADSENCTYFCGA NPYCNDLCTKNGAKSGYCQWVGKYGNGCW
BmPt2	91.25	1.62E-47	Venom protein	MKSAVIVAITLCCLFNLYANAQKDCSL PVDTGRGKGWFLRYYYNKNSKTCESFI YGGVGGNKNNFLNIENCCKICKAKNC
BmPt3	93.68	3.94E-59	Putative toxin-like peptide	MVKMQVIFIAFIAVIACSMVYGDSLSPWNEG DTYYGCQRQTDEFCNKICKLHLATGGGSC YQPFPFVKLCRCIGIDYDNSFFFGALEKQCPKLRE
BmPt4	78.75	1.77E-31	Venom peptide	AGRFDPVSAAGAGRFDPVLEAGAGRFDP ASAAGAGRFDPVSAAGAGRFDPTLEAG AGRFNPALEAGAGRFDPAAEAGAGRF
BmPt5	66.67	4.99E-33	Venom toxin	MFFLFLILLALIPMIKLEKECAMESLQGE FPRKDKGYLYECGEDIKCKKICKNHGRS ENDGKCCYGNCFCTGLHGKNIIKHQ

**Table 2:** New peptides/proteins with unknown function.

Name of Toxin	Sequnce
BmVt1	KIVVKYVKQRIADHIPRRNFKIHKRKFANNIYDSCII
BmVt2	AGRFDPALAAGARGYDPYYVGGPLGETKERIYIDE
BmVt3	MRALTIFGVIFACLLLFRTAVADHDGDDHNEELEEDVKQEKKILGPYSNMGRRTSCKIWIWQMEEAQRPS
BmVt4	IIHQIFQILLAALEKLNLMTKFLYLMIKAMIFLVEKLVVVEQNNVTWIHVRIMAIVMICGINTFVNVNDHFLE
BmVt5	MEKILLNQFIVFIVGIFGMRDVNSEYVSENFRKFPNYISENKDIQNKNNARNLIPDIKSSLKETETAESKKRNV DLINFSEKVKLSDESKKSFERTAVIHVLNNKENYDKNSKNTLNENLQPSSSNINKLKVTEISNNMNL
BmVt6	KVLEEEAGEGEEEEEENWQLMLQISVLFLYHKRDKIVYIIYDL
BmVt7	ILSNSKPLKSSRNNDELHKLFLNKNDNQPIKEFLGNVEKNNILSFPQNILPVYHPDFANMFSDEISFQTNTVP
BmVt8	KQEQDVLIQHRQQEQDVLTQYQQQERDVLIQH
BmVt9	MKVLVLLLVVALVAAALADKRDNIKRDYGGVGGGYGGGGRGGFGGGRGGFGG GYGGGRGGGIGGGRGGFGGGRGGFGGGRGGFGGGYGGGKYGGKYGGK
BmVt10	TMTFNRNYSLHHFRPCYVFIFLMSTIFWTLTNADEDNIKSTIDRDKRAPQLYSFGLGKKSYNIPVDSDNIES
BmVt11	TRSSTPERFSTRDLISSFSSASSVSSRCSAMSASEVCCCSRAMSWANWVRRRSSSPTRSLARCSSRSSI
BmVt12	MKCILINFLSLMLLANYSFGNEEQKKNMRALYPRKFYVERLRNDIVEEPFKKKNAYVTGG
BmVt13	MRVDNSAVLLVLIYLAKLSLSAPVDHGKEENPDPSFNIDVEYK NYLEEVARLLENDEDFINQIKDVTHSKDFQPEDISF
BmVt14	MRVVMLIFFLLCQALNLALVLQSEINFRYDSVETDLRSAELNNLDIWNED RKISRFLIEEEEISREISKYENAREISKRFLILVGTKWCGAGNKAE
BmVt15	RRGTAEIREGRFGISRRAEEADLRREPRYQPVYQHEVQQDSYGQNQDSLRQNNAAQQRSHYQTYDGQLGR
BmVt16	MKILSFIPVFLCLGIILNISCAENDLKRILNTSDEDLEKRWPDK
BmVt18	DPPECTCPHGKKFDHGLMKCIAGPEINCDSEIEGKGKTWTAFCEEENMVPCKHNIG DPPECTCPHGKKFDHGLMKCIAGPEINCDSEIEGKGKTWTAFCEEENMVPC
BmVt21	KDVNNLTYLIKKKLLEIIPKYKSPSIFSKSKMPIFPHENKESLFPKYKLQY
BmVt22	IILVGYCVKVMEDLVIAIFGNWHVGVMIFIIGFQLGLERPTNVVQNNTSIYVTF
BmVt23	MIKENMKKWLIYLLIPILSHSVVSSREGYRLNEQETYAADITPPHPQKHPYWYRRLWENQPPEQKDL YQNSYKNPFYLLDRKQLKNDDDEFELPNQAYLPLKEVGTNNRGADKIHSKSKIPEGHITVK
BmVt25	MKVVLVTFLCILLLLSNQNLGNDAGKKEIQAVYRRKAYADPAKNDDVEIVDHNFFRFRRSEELN
BmVt26	MKQFMFYFGIVIFATLLANRDVMAQNNKVILPGFIPEITQK
BmVt27	NSGLSLFGMKIIGYIFGFALICSLPNQNVCHENEGQKRSKIDAENDDLKQRVFPRVRFSLSDEEKRLQQR LSPAVLASLTGLEKQRLSRETLEDFSWNTEKRFDPAIFGSLPDDTKERIDSAALASLAENEEERFYSTT
BmVt29	LLIWSSLLPASPPSTKCVVFFFMPPLGEDSLKGQRKLFATLKFLPTV
BmVt30	VPSPPTPDAILELNEKLRDGRIWEFVHQRLVESPLLRAMVFNELTPRSSELNLDISNMRRRRSLQDYELK

## Supplementary Materials

The following are available online at www.mdpi.com/2072-6651/8/10/286/s1 (Tables S1 and S2).

## Abbreviations

The following abbreviations are used in this manuscript:

LC MS/MS	Liquid chromatography–mass spectrometry/mass spectrometry
2-DE	Two-dimensional gel electrophoresis
SDS-PAGE	Sodium Dodecyl Sulfate Polyacrylamide gel electrophoresis
RP-HPLC	Reversed-Phase High-Performance Liquid Chromatography
ESI-Q-TOF MS	Electrospray ionization tandem quadrupole/orthogonal-acceleration time-of-flight mass spectrometer
MALDI-TOF MS	Matrix-assisted laser desorption/ionization time-of-flight mass spectrometry
